# Image Encryption Based on Hopfield Neural Network and Bidirectional Flipping

**DOI:** 10.1155/2022/7941448

**Published:** 2022-02-11

**Authors:** Haitao Zhang, Shuangqi Yang

**Affiliations:** School of Software, Liaoning Technical University, Huludao 125105, China

## Abstract

Many encryption systems face two problems: the key has nothing to do with the plaintext; only a single chaotic sequence is adopted during the encryption. To solve the problems, this paper proposes an image encryption method based on Hopfield neural network and bidirectional flipping. Firstly, the plaintext image was segmented into blocks, the resulting image matrix was block scrambled, and each block was bidirectionally flipped to complete the scrambling process. After that, the plaintext image was processed by the hash algorithm to obtain the initial values and control parameters of the chaotic system, producing a pseudo-random sequence. Then, a diffusion matrix was generated through the optimization by Hopfield neural network and used to derive a ciphertext image through diffusion transformation. Experimental results show that our algorithm is highly sensitive to plaintext, strongly resistant to common attacks, and very efficient in encryption.

## 1. Introduction

The safety of digital images, an important carrier of information, has attracted much interest and concern [1]. To ensure the safety of image information, it is highly necessary to develop a good encryption algorithm [2]. The chaotic system lays a good foundation for encryption systems, due to its excellent sensitivity to initial values. Due to the sensitivity of the initial value, small changes can get completely different results, so each small change can achieve completely different encryption results during encryption, which can provide good security. As a result, the chaotic system is being integrated to more and more encryption algorithms. In the past two decades, researchers have proposed various encryption methods and applied them to image encryption, drawing on the unique properties of the chaotic system (e.g., sensitivity to initial values, unpredictability, and pseudo-randomness) and the natural bound between the system and cryptology [3–10]. Some scholars put forward several new chaotic systems and designed the corresponding encryption strategies [4–8]. Some scholars presented encryption algorithms based on existing chaotic systems, focusing on the design of encryption strategies [9–16]. Some scholars combined spatiotemporal chaos with DNA sequencing [6] and proposed image encryption algorithms based on the cryptological features of spatiotemporal nonadjacent coupled map lattices [17] and mixed linear-nonlinear coupled map lattices [18], respectively. The safety performance of an encryption algorithm can be measured by an important criterion: the ability to resist various attacks, namely, violent attack, statistical attack, and differential attack. Some algorithms are unable to withstand chosen-plaintext attack [19–24]. Besides, many algorithms are inefficient in encryption and need multiple encryptions to achieve a good effect. To solve the above defects, this paper explores key generation and image encryption strategy and proposes a chaotic image encryption algorithm based on Hopfield neural network and the image scrambling approach of bidirectional flipping. Firstly, the plaintext image was segmented into multiple *N* × *N* blocks, and the resulting image matrix was block scrambled. Each block was bidirectionally flipped and merged into a scrambled image. Next, the plaintext image was processed by the hash function, producing a hash array. On this basis, the control parameters and initial values of the chaotic system were determined to generate a random pseudo-matrix. Multiple sequences were taken as the initial conditions of Hopfield chaotic neural network, which creates the key flow of the diffusion matrix. Then, the scrambled image was segmented along the diagonal, and the key flow was converted into a key matrix. Afterwards, symmetric diffusion was performed on the key matrix and the scrambled image to obtain the encrypted image. Finally, the safety and reliability of our algorithm were demonstrated by comparing it with similar algorithms developed since 2017.

## 2. Image Encryption Strategy

### 2.1. Hopfield Neural Network

Proposed by American physicist Hopfield in 1982, the Hopfield neural network mimics the memory mechanism of biological neural networks. In this fully connected neural network, every node transmits a signal to other nodes, which eventually return the signal to the transmitter. Therefore, the Hopfield neural network has a feedback mechanism. A typical Hopfield neural network can be expressed as

(1) $$\begin{array}{c} x=-x_{i}+\sum_{i=1}^{3}w_{ij}v_{i}, \end{array}$$

where *v* is the hyperbolic tangent function:

(2) $$\begin{array}{c} v_{i}=\tanh\left(x_{i}\right)=\frac{e^{x_{i}}-e^{-x_{i}}}{e^{x_{i}}+e^{-x_{i}}}, \end{array}$$

and *w* is the weight function:

(3) $$\begin{array}{c} w=\left[\begin{array}{ccc} 2 & -1 & 0\\ 1.7 & 1.71 & 1.1\\ -2.5 & -2.9 & 0.56 \end{array}\right]. \end{array}$$

### 2.2. Encryption Flow

Our algorithm calls logistic mapping repeatedly:

(4) $$\begin{array}{c} x_{n+1}=rx_{n}\left(1-x_{n}\right), \end{array}$$

where *r* ∈ (0,4] is a control parameter. If 3.5699456 ≤ *r* ≤ 4, the logistic mapping will be chaotic; as *r* gradually approaches 4, the mapping becomes more and more chaotic and generates a chaotic sequence *x*_*n*_ of a better quality.

Taking an *M* × *M* plaintext image *P* for example, this paper designs a novel image encryption algorithm. There are three steps of the algorithm: image segmentation, block scrambling, and symmetric diffusion. After scrambling and diffusion, the plaintext image *P* is improved into a highly secure encrypted image.

## 3. Encryption Algorithm

Like most encryption strategies, our encryption strategy consists of two steps: scrambling and diffusion.

### 3.1. Bidirectional Flipping

Image scrambling aims to change the position of image pixels. The specific process of scrambling through block-based triangular transform is as follows.

Firstly, the original image is segmented into *N* × *N* blocks, each of which is subjected to triangular transform. The segmented image matrix consumes less resources in the process of computing encryption and can be further scrambled. Suppose the original image is of the size 256 × 256 and is broken down into 8 × 8 blocks. Then, the scrambling can be realized in the following steps:

*Step 1*. The hash function is applied on the plaintext image to generate a hash array. The relevant values are extracted from the array for initialization, producing the initial values and control parameters of the chaotic system. Then, a pseudo-random sequence is generated through the logistic chaotic system and is taken as the initial values of the Hopfield chaotic neural network. The optimal random sequence is thereby obtained.
*Step 2*. After segmentation, the image matrix is scrambled with the random sequence obtained by Hopfield chaotic neural network.
*Step 3*. The random sequence is numerically calculated. The rotation direction and angle of the blocks on the first layer of the image matrix are solved through remainder operation, with 90° as a unit. On this basis, the scrambling model is determined for the entire matrix. The calculation formulas are as follows:
  (5) $$\begin{array}{c} Z\left(i\right)=\text{floor}\left[\left(z\left(i\right)\times 10^{n}\right)\mathrm{mod}256\right],\\ H\left(i\right)=Z\left(i\right)\mathrm{mod}M. \end{array}$$
*Step 4*. Each image block is transformed into a matrix. The matrix of each block is rotated clockwise or counter-clockwise. As required by the algorithm, the adjacent layers are rotated in opposite directions.
*Step 5*. The scrambled block matrices are merged to a matrix as large as the original image matrix. This is the final result of image scrambling.

### 3.2. Image Diffusion

**Figure 1:**
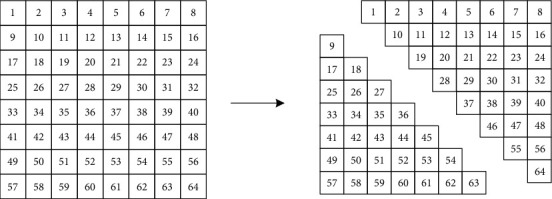
Diagonal segmentation model.

**Figure 2:**
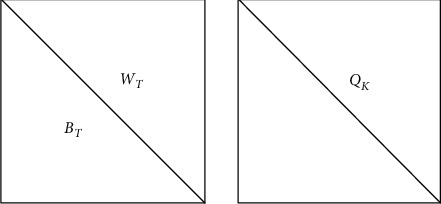
Schematic diagram of symmetrical segmentation.

Image diffusion mainly segments the original image matrix along the diagonal. The specific diffusion process is as follows:

*Step 1*. The initial state *x*_2_ and control parameter *r*_2_ of the logistic mapping are calculated as described in Section 2.1.
*Step 2*. Logistic mapping is implemented iteratively 200 times, producing chaotic sequences A1–A3. These sequences are imported to Hopfield chaotic neural network as initial parameters and converted into a diffusion key flow, forming a key matrix.
*Step 3*. The key matrix and scrambled image CC1 are segmented along the same direction. Suppose the matrix is of the size 8 × 8. The diagonal segmentation model is illustrated in Figure 1.
*Step 4*. After the diagonal segmentation model is determined, the diffusion is performed by (see Figure 2)
  (6) $$\begin{array}{c} \left\{\begin{array}{c} W_{T}^{\prime }\left(i,j\right)=W_{T}\left(i,j\right)⊕Q_{K}\left(i,j\right)\\ B_{T}^{\prime }\left(i,j\right)=B_{T}\left(i,j\right)⊕W_{T}^{\prime }\left(i,j\right) \end{array}\right.. \end{array}$$
*Step 5*. The diffusion image is obtained through the diffusion operation, and the final encrypted image *C* is outputted.

The decryption is the inverse process of encryption.

## 4. Simulation Results

To verify its effectiveness, our encryption algorithm was simulated on multiple images, using the simulation software GNU Octave. Through an experiment, the initial values of logistic mapping were determined as *x*_1_(0)=0.8761 and *x*_2_(0)=0.7323; the control parameters of logistic mapping were finalized as *r*_1_=3.9695 and *r*_2_=3.8925; the total number of iterations (TNI) was set to 200; different hash arrays *H* were generated from different plaintext images.

**Figure 3:**
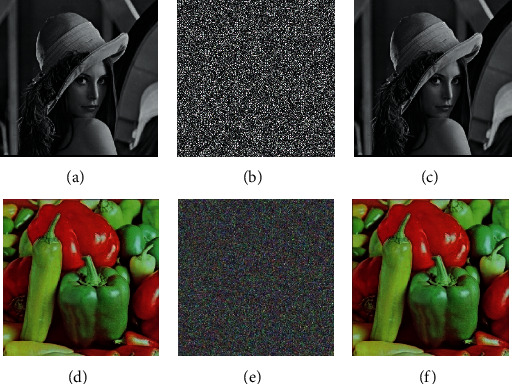
Simulation results. (a) Original image of Lena. (b) Encrypted image of Lena. (c) Decrypted image of Lena. (d) Original image of peppers. (e) Encrypted image of peppers. (f) Decrypted image of peppers.

Our simulation uses the gray image of Lena (256 × 256) and the color image of peppers. For the color image, firstly, the gray level of the color image is transformed, and the layer is divided into three different gray levels: R, G, and B, which are encrypted in the corresponding encryption process.  Figure 3 shows the generated encrypted images and decrypted images.

## 5. Safety Analysis

An ideal encryption algorithm should be able to resist various attacks, such as violent attack, statistical attack, and differential attack, and chosen-plaintext attack. To verify the safety of our algorithm, this paper theoretically analyzes and numerically simulates the algorithm in five aspects and compares it with the state-of-the-art chaotic theory-based algorithms [25–30].

### 5.1. Histogram Analysis

The ability of an encryption algorithm to resist statistical attack can be directly measured by the histogram of the ciphertext image, which describes the pixel distribution of the image. The statistical attack can easily steal some information from an image with uneven pixel distribution. Image itself is a form of data information, and image itself can be used as a carrier or directly as a kind of information transmission. The pixel distribution of the original image is distributed according to the content level of the image, so important content can be stolen and obstructed through statistical analysis attacks.

It can be known that the pixels were not evenly distributed on the plaintext histograms but evenly distributed on the ciphertext histograms. Therefore, the ciphertext images obtained by our encryption algorithm can resist the statistical attack.

### 5.2. Correlation Analysis

A high correlation between adjacent pixels indicates that the plaintext image is prone to the statistical attack. Thus, it is necessary for the encryption algorithm to reduce the correlation between adjacent pixels. 10,000 pixels were randomly selected from the plaintext and ciphertext images of the gray image of Lena, respectively. Then, the correlations between adjacent pixels in the horizontal, vertical, and diagonal directions were calculated by

(7) $$\begin{array}{c} r_{xy}=\frac{\mathrm{cov}\left(x,y\right)}{\sqrt{D\left(x\right)}\sqrt{D\left(y\right)}},\;\mathrm{cov}\left(x,y\right)=\frac{1}{N}\sum_{i=1}^{N}\left(x_{i}-E\left(x\right)\right)\left(y_{i}-E\left(y\right)\right),\\ D\left(x\right)=\frac{1}{N}\sum_{i=1}^{N}{\left(x_{i}-E\left(x\right)\right)}^{2},E\left(x\right)=\frac{1}{N}\sum_{i=1}^{N}x_{i}. \end{array}$$

In addition, the correlations between adjacent pixels in the ciphertext image of Lena obtained by our algorithm were compared with those in the ciphertext image of Lena obtained by other algorithms (Table 1).

### 5.3. Information Entropy Analysis

Information entropy is an important indicator of the randomness of information:

(8) $$\begin{array}{c} H\left(s\right)=\sum_{i=0}^{2^{n}-1}p\left(m_{i}\right)\log_{2}\frac{1}{p\left(m_{i}\right)}, \end{array}$$

where *p*(*s*_*i*_) is the probability of *s*_*i*_.

In theory, the probability of information leak decreases as the information entropy approaches 8.  Table 2 compares the information entropy of the cyphertexts of two test images obtained by our algorithm with that obtained by three other algorithms [25–27]. On both test images, the information entropy was approximately 8 in the ciphertexts obtained by our algorithm. The information entropy obtained by our algorithm was closer to 8 than that of any other algorithm. Therefore, the ciphertext images obtained by our algorithm are unlikely to suffer from information leak and are robust against the statistical attack.

### 5.4. Differential Attack

Differential attack is a kind of chosen-plaintext attack. During the attack, the attacker makes minor modifications to the plaintext image, encrypts the modified image and the original image separately, and compares the two encrypted images to find the correlations between plaintext and ciphertext images. The differential attack is commonly evaluated by the number of pixel change rate (NPCR) and the unified average changing intensity (UACI):

(9) $$\begin{array}{c} \text{NPCR}=\frac{1}{W\times H}\sum_{i=1}^{W}\sum_{j=1}^{H}D\left(i,j\right)\times 100\%,\\ \text{UACI}=\frac{1}{W\times H}\left(\sum_{i=1}^{W}\sum_{j=1}^{H}\frac{\left|c_{1}\left(i,j\right)-c_{2}\left(i,j\right)\right|}{255}\right)\times 100\%, \end{array}$$

where *W* and *H* are image width and height, respectively; *c*_1_ is the original plaintext image; and *c*_2_ is the plaintext image derived from  *c*_1_ by changing 1 bit of pixel value. If  *c*_1_(*i*, *j*) ≠ *c*_2_(*i*, *j*), then *D*(*i*, *j*)=1; otherwise, *D*(*i*, *j*)=0.

Theoretically, the result is good if NPCR and UACI approach 99.6093% and 33.4635%, respectively. Without changing the keys, our encryption algorithm was adopted to encrypt *c*_1_ and *c*_2_, respectively. Next, the NPCR and UACI were calculated for the two resulting ciphertext images. Tables 3 and 4 compare the NPCR and UACI of the ciphertexts obtained by our algorithm with those of the ciphertexts obtained by three other algorithms. Compared with those of other algorithms, the NPCR and UACI of our algorithm were mostly approximately 99.6093% and 33.4635%, respectively.

### 5.5. Robustness Analysis

**Figure 4:**
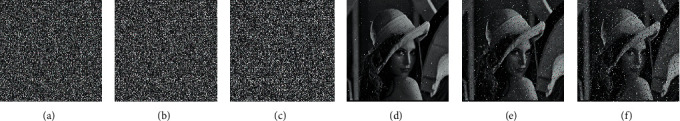
Noise test results. (a) Encrypted image of Lena with noise on the level of 0.01. (b) Encrypted image of Lena with noise on the level of 0.05. (c) Encrypted image of Lena with noise on the level of 0.1. (d) Decrypted image of Lena with noise on the level of 0.01. (e) Decrypted image of Lena with noise on the level of 0.05. (f) Decrypted image of Lena with noise on the level of 0.1.

Robustness is an important indicator of the anti-disturbance ability of a cryptosystem. Robustness analysis means that the decryption algorithm can still decrypt the content of the image even when the image is disturbed by other information and means, and the corresponding information can be obtained through the decrypted image, so as to prove the robustness of the algorithm. The robustness of our algorithm was tested by noise attack and denial-of-service (DoS) attack. Noise interference is an important issue in actual communication. Common noises include Gaussian noise, salt-and-pepper noise, etc. The salt-and-pepper noise stands out for its significant impact on ciphertext images. Therefore, this paper mainly tests the influence of the addition of salt-and-pepper noise to the plaintext image over our algorithm performance. Without changing the keys, different levels of salt-and-pepper noises were added to the plaintext image of Lena, and the noisy image was encrypted and decrypted by our algorithm.  Figure 4 shows the encrypted and decrypted images of the plaintext image of Lena with salt-and-pepper noise on the level of 0.01, 0.05, and 0.1, respectively. Even when the noise level was 0.1, the plaintext image could be distinguished in the image decrypted by our algorithm, evidencing the strong resistance of our algorithm to noise attack.

**Figure 5:**
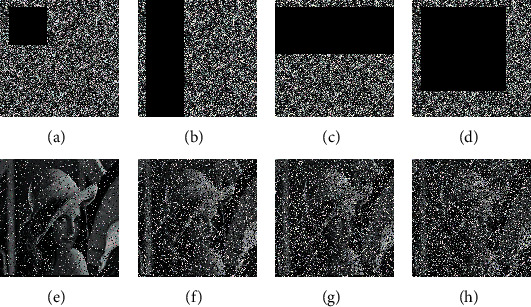
DoS attack test results. (a) Plaintext image of Lena with 6.25% of information being blocked. (b) Plaintext image of Lena with 25.44% of information being blocked. (c) Plaintext image of Lena with 40.20% of information being blocked. (d) Plaintext image of Lena with 87.89% of information being blocked. (e) Decrypted image of Lena with 6.25% of information being blocked. (f) Decrypted image of Lena with 25.44% of information being blocked. (g) Decrypted image of Lena with 40.20% of information being blocked. (h) Decrypted image of Lena with 87.89% of information being blocked.

The robustness of our algorithm was also tested against the DoS attack. Firstly, different portions of the information on the ciphertext image of Lena were blocked and then decrypted by our algorithm. Obviously, the quality of the decrypted image is negatively correlated with the amount of information being blocked.  Figure 5 presents the decrypted images of the ciphertext image of Lena with 6.25%, 25.44%, 40.20%, and 87.89% of information being blocked. It can be inferred that the main information of the plaintext image of Lena could still be recognized from the decrypted images. Therefore, our algorithm can effectively withstand the DoS attack and boasts strong robustness.

## 6. Conclusions

This paper mainly designs a chaotic image encryption algorithm based on Hopfield neural network and bidirectional flipping, a scrambling strategy. Firstly, the plaintext image was segmented into multiple blocks, and each block was scrambled. Then, SHA-512 hash algorithm was combined with the plaintext image to generate a hash array. On this basis, the initial values and control parameters were determined for the initialization of the chaotic mapping, and the control parameters were configured for scrambling and diffusion. Compared with other image encryption algorithms, our algorithm innovatively applies Hopfield neural network to encrypt images and adopts brand-new scrambling and diffusion models. Through simulation and theoretical analysis, it was confirmed that our algorithm is robust and effective in resisting statistical attack, differential attack, noise attack, and DoS attack.

## Figures and Tables

**Table 1:** Correlations between adjacent pixels in ciphertext image of Lena.

Image		Our algorithm	Wang et al.'s algorithm [28]	Farhan and Sanjeev's algorithm [29]	Hong et al.'s algorithm [30]
Lena	Horizontal	−0.0016	−0.0031	−0.0146	0.0020
	Vertical	0.0043	0.0084	0.0098	0.0042
	Diagonal	−0.0026	−0.0007	0.0056	0.0013

**Table 2:** Comparison of information entropy of ciphertext images.

Image	Our algorithm	Wang and Guan's algorithm [25]	Niyat et al.'s algorithm [26]	Wu et al.'s algorithm [27]
Lena	7.9988	7.9976	7.9974	7.9976
Peppers	7.9994	7.9980	7.9972	7.9974

**Table 3:** Comparison of NPCR (%) of ciphertext images.

Image	Our algorithm	Wang et al.'s algorithm [28]	Farhan and Sanjeev's algorithm [29]	Hong et al.'s algorithm [30]
Lena	99.6231	99.6016	99.6356	99.6037
Peppers	99.6307	99.6091	99.5891	99.6124

**Table 4:** Comparison of UACI (%) of ciphertext images.

Image	Our algorithm	Wang et al.'s algorithm [28]	Farhan and Sanjeev's algorithm [29]	Hong et al.'s algorithm [30]
Lena	33.4463	33.4735	33.4147	33.4381
Peppers	33.4768	33.6234	33.3568	33.7216

## Data Availability

The data used to support the findings of this study are available from the corresponding author upon request.
